# Long-Range Surface-Directed Polymerization-Induced Phase Separation: A Computational Study

**DOI:** 10.3390/polym13020256

**Published:** 2021-01-14

**Authors:** Shima Ghaffari, Philip K. Chan, Mehrab Mehrvar

**Affiliations:** Department of Chemical Engineering, Ryerson University, 350 Victoria Street, Toronto, ON M5B 2K3, Canada; shima.ghaffari@ryerson.ca (S.G.); mmehrvar@ryerson.ca (M.M.)

**Keywords:** long-range surface potential, polymerization-induced phase separation, surface-directed spinodal decomposition, wetting layer

## Abstract

The presence of a surface preferably attracting one component of a polymer mixture by the long-range van der Waals surface potential while the mixture undergoes phase separation by spinodal decomposition is called long-range surface-directed spinodal decomposition (SDSD). The morphology achieved under SDSD is an enrichment layer(s) close to the wall surface and a droplet-type structure in the bulk. In the current study of the long-range surface-directed polymerization-induced phase separation, the surface-directed spinodal decomposition of a monomer–solvent mixture undergoing self-condensation polymerization was theoretically simulated. The nonlinear Cahn–Hilliard and Flory–Huggins free energy theories were applied to investigate the phase separation phenomenon. The long-range surface potential led to the formation of a wetting layer on the surface. The thickness of the wetting layer was found proportional to time t*^1/5^ and surface potential parameter *h*_1_^1/5^. A larger diffusion coefficient led to the formation of smaller droplets in the bulk and a thinner depletion layer, while it did not affect the thickness of the enrichment layer close to the wall. A temperature gradient imposed in the same direction of long-range surface potential led to the formation of a stripe morphology near the wall, while imposing it in the opposite direction of surface potential led to the formation of large particles at the high-temperature side, the opposite side of the interacting wall.

## 1. Introduction

The surface-directed spinodal decomposition (SDSD) phenomenon occurs where a surface preferentially adsorbs one component of a binary mixture undergoing phase separation by spinodal decomposition. The SDSD leads to the formation of a wetting layer enriched by the preferred component adjacent to the surface and propagation of the anisotropic concentration waves perpendicular to the wall into the bulk, and then a crossover to the isotropic droplet-type or interconnected morphology formed by the phase separation via spinodal decomposition in the bulk [1,2,3]. The SDSD can improve the physical, mechanical, and surface characteristics of the polymer materials by developing layered morphologies [3,4,5,6,7,8,9,10,11,12]. On the other hand, it may lead to the formation of undesirable enrichment layer(s) and adversely affect the mechanical and thermal properties of polymer materials [7,13]. Therefore, it is significant to understand and control the SDSD, as it has technological applications in the food industry [8], as well as the formation of polymer blends [3,4,9,10,11,12] and photovoltaic devices [7].

When a polymer–solvent mixture undergoing phase separation is confined between the walls that preferably attract the solvent, the translational symmetry in the direction normal to the wall surface is broken and results in the formation of a partially wetting (PW) or completely wetting (CW) layer on the wall surface [14]. The relative surface tensions between the solvent-rich (S-rich) phase, polymer-rich (P-rich) phase, and surface(s) determine whether the completely wet equilibrium morphology or the partially wet morphology is achieved. When a PW layer forms, there is a contact angle (*θ*) of the polymer-rich and solvent-rich phases with the wall surface. Young’s condition determines the contact angle as *σ*cos*θ* = *γ*_Ps_ − *γ*_Ss_, where *σ* is the polymer–solvent interfacial tension, *γ*_Ps_ is the P-rich phase–surface interfacial tension, and *γ*_Ss_ is the S-rich phase–surface interfacial tension. If *γ*_Ps_ − *γ*_Ss_ > *σ*, the P-rich phase is completely removed from the wall surface and a CW layer enriched by the S-rich phase forms on the wall. The interface of the P-rich phase–S-rich phase is parallel to the wall surface in the CW condition [14].

The investigation on the surface-directed spinodal decomposition of the polymer blends illustrated that the long-range van der Waals forces led to the development of different morphologies from those achieved under the short-range surface potential [3]. Comprehensive numerical studies on the long-range surface-directed phase separation of polymer mixtures by considering the long-range van der Waals forces were carried out [13,15,16,17,18,19,20,21,22].

Chen et al. [15] investigated the long-range surface-directed phase separation of polymer *A*–*B* mixture, where polymer *A* was preferably attracted to the wall surface, using the mean-field theory. They found that the long-range surface interactions compared to the short-range surface effect led to a slower decrease of volume fraction of polymer *A ϕ_A_* versus distance from the wall *z*, where *z* is small. Farther from the wall (*z* ≫ 1), the decay of volume fraction was found proportional to *z*^−3^, while it decayed exponentially in the short-range wetting transition. In the region of small and intermediate *z*, the volume fraction of polymer *A* was more extensive than that under the short-range interactions [15].

Brown and Chakrabarti [16] developed a two-dimensional model for the long-range surface-directed phase separation of a binary mixture under critical quenches. The surface-interaction potential *V* was defined as −*σ* for *y* = 0, and −*σ*/*y*^(*p*+1)^ for *y* > 0, where *σ* = 0.4, and *p* = 1 and 2. The thickness of the surface enrichment layer and the average characteristic domain size in parallel and perpendicular directions to the surface were proportional to *t*^1/3^, where *t* is time. However, at each time, the average domain size in the parallel direction was found larger compared to that in the perpendicular direction. The correlation function and density profile versus the scaled distance from the wall in both directions at late times obeyed the dynamical scaling trend well. The density profiles demonstrated the characteristic oscillations close to the wall surface, which decayed by moving into the bulk [16]. They also numerically investigated the ordering of both symmetric and asymmetric block copolymers quenched in the presence of long-range surface interactions [17]. The surface potential *V* and the values of *σ* and *p* were considered the same as those in [16]. For the symmetric compositions, the equilibrium thickness of the wetting layer depended on the copolymer chain length (*N*) by a power-law function where the exponent was found to be 0.6. The off-critical quench of asymmetric copolymer melt into the unstable region of the phase diagram led to the formation of layered morphology close to the surface, while circular droplets were observed in the bulk [17]. 

The phase separation of binary polymer blends confined between the walls, in which one of them preferentially attracted one component, was investigated numerically by Binder et al. [18]. For the critical quench cases, the effects of long-range surface-directed spinodal decomposition on the order parameter profile and evolution of wetting layer thickness and pair length scales were investigated. The long-range surface potential (*V*) was defined as *V* = *h*_1_/*Z^n^*, where *Z* is the distance from the interacting wall, and *h*_1_ and *n*, respectively, are the surface potential parameter and exponent. For *h*_1_ = 8 and *n* = 2, the evolution of the wetting layer occurred much faster compared to that under the short-range interactions. The length scales in both parallel and perpendicular directions to the surface, respectively, *L*_||_ and *L*_┴_, grew with time based on a power-law function, where the exponent was found as 1/3 (for *n* = 3 and *h*_1_ = 8) at earlier times. At later times, a crossover happened, and the exponent increased to 1/2 for the *L*_||_, while the growth of *L*_┴_ slowed down. The thickness of the wetting layer was found proportional to the power of time, where the exponent was 0.16 (for *n* = 3 and *h*_1_ = 12) [18].

Puri and Binder [19,20,21] carried out comprehensive computational studies on the surface-directed phase separation of polymer mixtures with various initial compositions undergoing off-critical quenches. In a semi-infinity geometry, the long-range surface potential as defined above (*V*(*z*) = *h*_1_/*z^n^*) led to the time evolution of thickness of surface enrichment layer *R*_1_ by power-law function (*R*_1_(*t*)∼*t^m^*). For *ψ*_0_ ≤ 0, where *ψ*_0_ is the difference of composition field from the critical composition, the slope of ln *R*_1_ vs. ln *t*, which defined *m*, was obtained. For *n* = 4, the slope was found to be 1/6 for *t* < *t*_c_ and 1/3 for *t* > *t*_c_, where *t*_c_ is the time at which the crossover occurred. They found the following correlation for the growth rate of the enrichment layer: *R*_1_(*t*)∼(*h*_1_*t*)^1/(*n*+2)^, where *t* < *t*_c_. For *ψ*_0_ > 0 and *n* = 4, the exponent of the wetting layer growth rate *m* was obtained equal to 1/6.

Xie and Yan [22] presented a three-dimensional model using the cell dynamic systems (CDS) to investigate the effect of long-range surface potential on the morphology development of binary polymer mixtures undergoing off-critical quenches. According to their findings, the thickness of the wetting substrate *R* was proportional to *t*^1/(*n*+2)^ and *H_a_*^1/(*n*+2)^ before the crossover where *n* is the exponent of surface potential and *H_a_* is the surface potential parameter; after the time in which the crossover occurred, *R* was found to be independent to *H_a_*, while it was related to time *t* based on the Lifshitz–Slyozov power law.

Tabatabaieyazdi et al. [13] simulated the long-range surface-directed phase separation of polymer mixtures under the temperature gradients. The analysis of structure factor for the bulk illustrated the exponential and Lifshitz–Slyozov power-law growths in the early and intermediate stages of phase separation, respectively. The thickness of the wetting layer was not noticeably affected by changing the values of temperature gradients. A semi-wetting layer was not observed for the range of temperature gradients imposed. A deeper quench depth led to a faster phase separation and the formation of a thinner enrichment layer.

The studies carried out so far on the long-range surface-directed spinodal decomposition are based on the thermal-induced phase separation technique, in which the polymer mixture is thermally quenched into the unusable region of the phase diagram and phase separation is induced. However, there has been no study on the long-range surface-directed polymerization-induced phase separation published in the literature according to the authors’ knowledge. In long-range surface-directed polymerization-induced phase separation, as the polymerization proceeds, it simultaneously induces the phase separation by spinodal decomposition while a wall surface is preferentially attracting one of the components of the mixture by the long-range surface potential.

The conventional polymerization-induced phase separation method in which no external gradient is imposed to the system and the morphology is isotropic has been extensively investigated. Macosko and his coworkers [23,24,25,26,27,28,29] carried out comprehensive experimental studies on the polymerization-induced phase separation (PIPS) approach to fabricate the heterogeneous systems of reaction injection-molded (RIM) polyurethanes, polyureas, polyarylate (PAR) and ethylene-ethyl acrylate glycidyl methacrylate (E-EA-GMA) blends, protein-based hydrogels, and copolymers. 

Szczepanski et al. [30] investigated the photo-polymerization of triethylene glycoldimethacrylate (TEGDMA) modified by poly(methyl methacrylate) (PMMA), which induced the phase separation and formed the heterogeneous network. The obtained tan delta profile showed the formation of two separated phases, one rich in TEGDMA, and the other rich in TEGDMA/PMMA.

Kim et al. [31] applied the PIPS approach to produce the monodisperse micro-capsules with size-selective permeability using a microfluidic procedure. The applications of semipermeable microcapsules include the controlled release of drugs, the study of cell-to-cell communication, and the isolation of enzymes or artificial catalysts. In a capillary microfluidic device, monodisperse water-in-oil-in-water (W/O/W) double-emulsion drops consisting of photocurable resin and inert oil in their ultrathin middle layer were created using UV irradiation. The monomers were photopolymerized under UV illumination, so phase separation occurred between the polymerized resin and the oil. Then, the removal of porogen oil created regular pores in the polymerized membrane, which linked the interior and exterior of the microcapsules by means of size-selective permeability. The degree of phase separation was adjusted by regulating the fraction of oil in the shell or the affinity of the oil to the monomers.

The macrophase separation occurring during the free-radical copolymerization of styrene and dimethacrylate in the presence of poly (methyl methacrylate) (PMMA) as a modifier was investigated by Schroeder et al. [32]. The results obtained by the scanning electron microscopy (SEM) and the real-time static light scattering (LS) showed no polymerization-induced phase separation occurred without adding PMMA; PMMA did not undergo the polymerization, but it induced the phase separation.

Fabrication of the anisotropic polymer blends by photopolymerization under a UV light intensity gradient was experimentally investigated by Fujiki et al. [33]. The anisoptroy was achieved by irradiation from one side of the mixture undergoing the photopolymerization. Our research group theoretically investigated the polymerization-induced phase separation of the polymer solutions without any external gradient, and also under the temperature gradient, concentration gradient, and short-range surface potential [34,35,36,37,38]. In addition, the short-range, long-range, and multiple surface-directed thermal-induced phase separation of the polymer blends were studied [13,39,40]. However, the polymerization-induced phase separation of the polymer solutions under a long-range surface potential was not simulated. Therefore, in this study, the long-range surface-directed polymerization-induced phase separation of a monomer–solvent mixture, which was initially homogeneous, was theoretically studied. The monomer underwent self-condensation polymerization, which led to the upward movement of the phase diagram toward higher solvent concentration; the phase diagram eventually crossed the curing point placing sample in the unstable region of the phase diagram, causing phase separation to occur. In the meantime, the wall surface at x* = 0 preferentially attracted the solvent as a result of long-range surface potential. The model could predict well the development of droplet-type morphology in the bulk as well as the surface enrichment layer on the wall. The effects of various parameters on the surface-directed phase-separated structure were investigated and presented in this manuscript.

## 2. Model Development 

In the current section, a two-dimensional model of the polymerization-induced phase separation of a solvent–polymer mixture with long-range surface potentials at the wall surface at x* = 0 attracting the solvent is developed. The initial miscible mixture consists of a solvent and a tri-functional monomer *A*_3_, which undergoes self-condensation polymerization, and as a result, the phase separation of solvent–polymer mixture by spinodal decomposition is induced. 

The nonlinear Cahn–Hilliard (C-H) theory, which describes the concentration spatio-temporal distribution of the binary mixtures undergoing the phase separation by spinodal decomposition, is derived from the continuity equation [41]:

(1) $$\frac{\partial c}{\partial t}=-\nabla \cdot j$$

where *c* is the solvent concentration, which describes the volume fraction of the solvent in this study, *t* is time, and **j** is the interdiffusional flux.

The interdiffusional flux **j** is related to the chemical potential gradient of two components as:

(2) $$j=-M\nabla (\mu_{2}-\mu_{1})=-M\nabla (\frac{\delta F}{\delta c})$$

where *M* is the mobility of the mixture, *μ*_1_ and *μ*_2_ represent the chemical potentials of the components, and *F* is the bulk total free energy of the heterogeneous binary mixture. 

For the long-range surface-directed spinodal decomposition, *F* is described according to the following modified equation [13,15,16]:

(3) $$F=\int \left[f\left(c\right)+\kappa {\left(\nabla c\right)}^{2}\right]dV+\int \left[k_{B}TV\left(x\right)c\right]dx$$

where *f*(*c*) is the free energy density of the homogeneous blend, *κ* is the interfacial energy coefficient, *k_B_* is the Boltzmann’s constant, and *T* is the temperature. The term containing *V*(*x*) corresponds to the long-range surface-interaction potential contributing to the bulk total free energy. *V*(*x*) is a function with which the surface potential decays by moving away from the surface in the *x*-direction.

Combining Equations (1)–(3), the kinetic equation of long-range surface-directed phase separation by spinodal decomposition of the mixture becomes:

(4) $$\frac{\partial c}{\partial t}=\nabla \cdot [M\nabla [\frac{\partial f}{\partial c}-2\kappa \nabla^{2}c+k_{B}TV]]$$

which represents the nonlinear C-H equation with an added term to take into account the long-range surface effect.

The Flory–Huggins free-energy equation is used in this study, so *f*(*c*) is expressed as follows [42]:

(5) $$f(c)=\frac{k_{B}T}{\nu }\left[\frac{c}{N_{1}}\ln c+\frac{\left(1-c\right)}{N_{2}}\ln(1-c)+\chi c(1-c)\right]$$

where *ν* is the volume of a cell, and *N*_1_ and *N*_2_ represent the degrees of polymerization of solvent and solute, respectively. In the current study, the degree of polymerization of the solvent is considered equal to 1 (*N*_1_ = 1). *χ* is Flory’s interaction parameter, which is assumed to be a function of temperature only [43]:

(6) $$\chi =\frac{1}{2}-\psi [1-\frac{\theta }{T}]$$

where *ψ* is the dimensionless entropy of the dilution parameter and *θ* shows the theta temperature. 

Mobility *M* is a function of the self-mobilities of components, which is presented based on the slow-mode theory considering that the slower moving component controls the binary diffusion [44]:

(7) $$\frac{1}{M}=\frac{1}{M_{1}}+\frac{1}{M_{2}}$$

The self-mobility of each component *M_i_* is expressed as a function of its self-diffusion coefficient *D_i_*, and in turn, its degree of polymerization *N_i_*, according to the following equations [44]:

(8) $$D_{i}=M_{i}(\frac{\partial^{2}f}{\partial c_{i}{}^{2}})$$

(9) $$D_{i}=\frac{k_{B}T}{\xi_{i}N_{i}}\;\mathrm{for}\;i=1,\;2$$

Equation (9) was derived from the Rouse theory, which is appropriate for short-chain polymers with *N*_2_ < 200, as the impact of entanglement of polymer chains is ignored in the theory. *ξ_i_* presents the frictional coefficient per cell of the solvent (*i* = 1) or polymer molecule (*i* = 2). Assuming that the frictional coefficients of solvent and polymer segments are equal (*ξ*_1_ = *ξ*_2_ = *ξ*), and independent of pressure and temperature, the mobility is expressed as [44]:

(10) $$M=\frac{\nu c(1-c)}{\xi }$$

The interfacial parameter *κ* depends on the molecular weight of the polymer, and as a result, the degree of polymerization *N*_2_ as follows [34]:

(11) $$\kappa =\kappa_{0}N_{2}$$

where *κ*_0_ is the interfacial energy parameter of the monomer. Equation (11) is applicable to the short-chain polymer solutions. 

The sample consists of a solvent (*N*_1_ = 1) and a tri-functional monomer undergoing self-condensation polymerization. Since the self-condensation polymerization is a second-order reaction, the kinetic rate of polymerization is found as [34]:

(12) $$\frac{dp}{dt}=k_{1}{\left(1-p\right)}^{2}$$

where *p* and *k*_1_ are the extent of reaction and the polymerization rate constant, respectively. The extent of reaction can be obtained by solving Equation (12) analytically as:

(13) $$p=\frac{k_{1}t}{1+k_{1}t}$$

Applying the Arrhenius equation, *k*_1_ depends on the temperature as follows:

(14) $$k_{1}=A\exp(\frac{-E_{a}}{RT})$$

where *A* is the collision frequency factor, *E_a_* is the activation energy, and *R* is the gas constant.

The degree of polymerization of solute is expressed based on the weight average degree of polymerization as follows [34]:

(15) $$N_{2}=\frac{1+\alpha }{1-\alpha (f-1)}$$

*α* represents the branching coefficient, which is equal to the extent of reaction (*α* = *p*) for the single reactant *A*_3_. *f* is the functionality of monomer, which *f* = 3 in this study.

Combining Equations (13)–(15), the time and temperature dependence of the degree of polymerization is expressed by the following equation:

(16) $$N_{2}=\frac{1+2At\exp(\frac{-E_{a}}{RT})}{1-At\exp(\frac{-E_{a}}{RT})}$$

In some cases, a linear temperature gradient in the *x*-direction is imposed:

(17) $$T=({\frac{T_{2}-T_{1}}{x_{2}-x}}_{1})(x-x_{1})+T_{1}$$

*T*_1_ and *T*_2_ show the temperature at positions *x*_1_ and *x*_2_, respectively. 

All the variables and parameters are rescaled into the following dimensionless relations:

(18) $$x^{*}=\frac{x}{L}\;(\mathrm{Dimensionless}\;\mathrm{length}\;\mathrm{of}\;\mathrm{the}\;\mathrm{domain})$$

(19) $$y^{*}=\frac{y}{L}\;(\mathrm{Dimensionless}\;\mathrm{length}\;\mathrm{of}\;\mathrm{the}\;\mathrm{domain})$$

(20) $$T^{*}=\frac{T}{\theta }\;(\mathrm{Dimensionless}\;\mathrm{temperature})$$

(21) $$c^{*}=c\;(\mathrm{Dimensionless}\;\mathrm{concentration})$$

(22) $$E_{a}^{*}=\frac{E_{a}}{R\theta }\;(\mathrm{Dimensionless}\;\mathrm{activation}\;\mathrm{energy})$$

(23) $$A^{*}=\frac{AL\xi }{2\kappa_{0}\nu }\;(\mathrm{Dimensionless}\;\text{pre-exponential}\;\mathrm{factor})$$

(24) $$t^{*}=\frac{2\nu \kappa_{0}t}{\xi L^{4}}\;(\mathrm{Dimensionless}\;\mathrm{time})$$

(25) $$D=\frac{k_{B}\theta L^{2}}{2\kappa_{0}\nu }\;(\mathrm{Dimensionless}\;\mathrm{diffusion}\;\mathrm{coefficient})$$

(26) $$K^{*}=A^{*}t^{*}\exp(\frac{-E_{a}^{*}}{T^{*}})\;(\mathrm{Dimensionless}\;\mathrm{rate}\;\mathrm{constant})$$

(27) $$N_{2}=\frac{1+2K^{*}}{1-K^{*}}\;(\mathrm{Dimensionless}\;\mathrm{degree}\;\mathrm{of}\;\mathrm{polymerization})$$

(28) $$V^{*}\left(x^{*}\right)=\left\{ \begin{array}{ll} h_{1}, & x^{*}=0\\ \frac{h_{1}}{{\left(\sigma x^{*}\right)}^{n}}, & x^{*}>0 \end{array}\right.\;(\mathrm{Dimensionless}\;\text{long-range}\;\mathrm{surface}\;\mathrm{potential})$$

Furthermore, the domain is a square with the dimensions *L* × *L*. *h*_1_ is the surface potential parameter showing the strength of the long-range surface forces, and *σ* and *n*, respectively, represent the coefficient and exponent with which the long-range potential decays in the bulk. 

Combining Equations (4), (5), (10), (11), (16) and (18)–(28), the following dimensionless nonlinear fourth-order partial differential equation is achieved to predict the spatio-temporal concentration profile of the solvent when the long-range surface-directed polymerization-induced phase separation at a constant temperature is occurring:

(29) $$\begin{array}{l} \frac{\partial c^{*}}{\partial t^{*}}=DT^{*}\left[\frac{1}{N_{2}}-1-2\chi \left(1-2c^{*}\right)\right]\nabla^{*}c^{*}\cdot \nabla^{*}c^{*}+DT^{*}\left[1-c^{*}+\frac{c^{*}}{N_{2}}-2\chi c^{*}\left(1-c^{*}\right)\right]\nabla^{*2}c^{*}\\ +DT^{*}\left(1-2c^{*}\right)\nabla^{*}c^{*}\cdot \nabla^{*}V^{*}+DT^{*}c^{*}\left(1-c^{*}\right)\nabla^{*}{}^{2}V^{*}-N_{2}\left(1-2c^{*}\right)\nabla^{*}c^{*}\cdot \nabla^{*}{}^{3}c^{*}-N_{2}c^{*}\left(1-c^{*}\right)\nabla^{*}{}^{4}c^{*} \end{array}$$

Equations (4)–(6), (10), (11), (16)–(28) are incorporated to obtain the following partial differential equation, which describes the spatio-temporal dimensionless concentration distribution of the solvent when a linear spatial temperature gradient is imposed to the sample.

(30) $$\begin{array}{l} \frac{\partial c^{*}}{\partial t^{*}}=D\left[\frac{c^{*}\left(1-c^{*}\right)\left[\ln\left(1-c^{*}\right)+1\right]\left(E_{a}^{*}fK^{*}\right)}{T^{*}{}^{2}\left(1+2K^{*}\right)}\right]\nabla^{*}T^{*}\cdot \nabla^{*}T^{*}\\ +D\left[\frac{7}{2}-8c^{*}+4c^{*}{}^{2}-\psi +8\psi c^{*}\left(1-c^{*}\right)+\left(1-2c^{*}\right)\ln c^{*}-\frac{\left(1-2c^{*}\right)\ln\left(1-c^{*}\right)+1-4c^{*}}{N_{2}}\right]\nabla^{*}c^{*}\cdot \nabla^{*}T^{*}\\ +D\left[\frac{\left[\left(1-2c^{*}\right)\ln\left(1-c^{*}\right)+1-4c^{*}\right]\left(E_{a}^{*}fK^{*}\right)}{T^{*}\left(1+2K^{*}\right)}\right]\left[\frac{E_{a}^{*}\left(1-2K^{*}+fK^{*}\right)}{T^{*}\left(1+2K^{*}-fK^{*}\right)}-\frac{2E_{a}^{*}fK^{*}}{T^{*}\left(1+2K^{*}\right)\left(1+2K^{*}-fK^{*}\right)}\right]\nabla^{*}c^{*}\cdot \nabla^{*}T^{*}\\ +D\left(1-2c^{*}\right)V^{*}\nabla^{*}c^{*}\cdot \nabla^{*}T^{*}+2Dc^{*}\left(1-c^{*}\right)\nabla^{*}V^{*}\cdot \nabla^{*}T^{*}+DT^{*}\left[\frac{1}{N_{2}}-1-2\chi \left(1-2c^{*}\right)\right]\nabla^{*}c^{*}\cdot \nabla^{*}c^{*}\\ +DT^{*}\left[1-c^{*}+\frac{c^{*}}{N_{2}}-2\chi c^{*}\left(1-c^{*}\right)\right]\nabla^{*}{}^{2}c^{*}+DT^{*}\left(1-2c^{*}\right)\nabla^{*}c^{*}\cdot \nabla^{*}V^{*}+DT^{*}c^{*}\left(1-c^{*}\right)\nabla^{*}{}^{2}V^{*}\\ -\left(1-2c^{*}\right)\left(\frac{\partial N_{2}}{\partial T^{*}}\right)\nabla^{*}{}^{2}c^{*}\nabla^{*}c^{*}\cdot \nabla^{*}T^{*}-c^{*}\left(1-c^{*}\right)\left(\frac{\partial^{2}N_{2}}{\partial T^{*}{}^{2}}\right)\nabla^{*}{}^{2}c^{*}\nabla^{*}T^{*}\cdot \nabla^{*}T^{*}-2c^{*}\left(1-c^{*}\right)\left(\frac{\partial N_{2}}{\partial T^{*}}\right)\nabla^{*}T^{*}\cdot \nabla^{*}{}^{3}c^{*}\\ -N_{2}\left(1-2c^{*}\right)\nabla^{*}c^{*}\cdot \nabla^{*}{}^{3}c^{*}-N_{2}c^{*}\left(1-c^{*}\right)\nabla^{*}{}^{4}c^{*} \end{array}$$

To solve Equations (29) and (30), first, the initial and boundary conditions are determined. The initial homogeneous mixture contains the infinitesimal concentration fluctuations; therefore, the initial condition is specified as:

(31) $$c^{*}\left(t^{*}=0\right)=c_{0}{}^{*}+\delta c^{*}\left(t^{*}=0\right)$$

where *c*_0_* expresses the dimensionless initial average concentration of the solvent, and δc* represents the dimensionless concentration fluctuations, which is random values in the range of ±10^−6^ in this study.

The three sets of boundary conditions used in this study are as follows:

1. The zero mass flux boundary condition [45,46]:

(32) j=0

which for a square geometry is expressed as:

(33) $$\frac{\partial^{3}c^{*}}{\partial x^{*}{}^{3}}+\frac{\partial^{3}c^{*}}{\partial x^{*}\partial y^{*}{}^{2}}=0,\;\mathrm{at}\;t^{*}>0,\;\mathrm{and}\;x^{*}=0\;\mathrm{and}\;x^{*}=1$$

(34) $$\frac{\partial^{3}c^{*}}{\partial y^{*}{}^{3}}+\frac{\partial^{3}c^{*}}{\partial y^{*}\partial x^{*}{}^{2}}=0,\;\mathrm{at}\;t^{*}>0,\;\mathrm{and}\;y^{*}=0\;\mathrm{and}\;y^{*}=1\;\;$$

2. The natural boundary condition [45,46]:

(35) $$[\nabla^{*}c^{*}]\cdot n=0$$

which is valid at the walls without any surface forces and expressed as:

(36) $$\frac{\partial c^{*}}{\partial x^{*}}=0,\;\mathrm{at}\;t^{*}>0,\;\mathrm{and}\;x^{*}=1$$

(37) $$\frac{\partial c^{*}}{\partial y^{*}}=0,\;\mathrm{at}\;t^{*}>0,\;\mathrm{and}\;y^{*}=0\;\mathrm{and}\;y^{*}=1,$$

3. The surface potential boundary condition [47]:

(38) $$\frac{\partial c^{*}}{\partial t^{*}}=\frac{\delta F_{s}^{*}}{\delta c^{*}}=-h_{1}-gc^{*}+\gamma \frac{\partial c^{*}}{\partial x^{*}},\;\mathrm{at}\;t^{*}>0,\;\mathrm{and}\;x^{*}=0$$

which is applied to x* = 0 where the wall surface attracts the solvent. *h*_1_, *g*, and γ are the parameters representing the surface phase diagram, and F_s_* is the dimensionless surface free energy, which is obtained as [48]:

(39) $$F_{s}^{*}=\int {\left[-h_{1}c^{*}-\frac{g}{2}c^{*2}+\gamma c^{*}\frac{\partial c^{*}}{\partial x^{*}}\right]}_{x^{*}=0}dy^{*}$$

The static form of the surface potential boundary condition is considered in this study, since the concentration of the solvent at the wall surface at x* = 0 reaches its equilibrium value much faster compared to the time scales of phase separation by spinodal decomposition [21,48,49,50]. Therefore, the temporal derivative of the solvent concentration on the wall surface in Equation (38) is ignored, and the surface potential boundary condition is expressed as [13,39,40]:

(40) $$\frac{\partial c^{*}}{\partial x^{*}}=\frac{h_{1}}{\gamma }+\frac{g}{\gamma }c^{*},\;\mathrm{at}\;t^{*}>0,\;\mathrm{and}\;x^{*}=0$$

The Galerkin finite element method is applied to solve the governing equations numerically. A mesh of 100 × 100 nodes is used to discretize the square domain. Applying the finite element method, a set of time-dependent ordinary differential equations is achieved and solved by the Newton–Raphson iterative method. The Forward Euler–Backward Euler method is applied to solve the time integration; convergence is obtained when the difference between two consecutive solutions is less than 10^−6^. The C^++^ programming language was used to carry out the simulations; the C^++^ codes were executed on the Workstation (256G/2 Processors, 18-Core each) in the graduate computer lab of the Chemical Engineering Department, Ryerson University; each run took 8–24 h.

## 3. Results and Discussion

Although a comprehensive study was performed by varying the parameter values in the mathematical models outlined above, the six case studies presented here fulfill the objectives of this paper. The effects of various parameters such as the surface potential parameter, the surface potential exponent, the diffusion coefficient, and the temperature gradient, on the growth of the wetting layer on the wall and the droplets in the bulk were extensively investigated. The parameters applied to the simulations are consistent with the experimental values reported in the literature for the temperature *T* [51], time *t* [52], degree of polymerization [29], reaction rate constant *k*_1_ [53], diffusivity *D* [29], and length *L* [39]. The conditions and parameters applied to each case are listed in Table 1.

**Figure 1:**
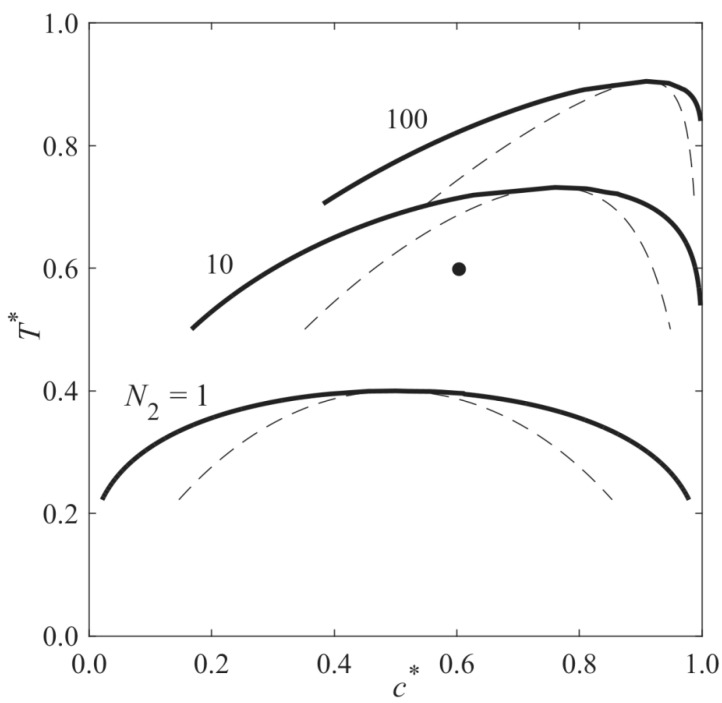
Phase diagram of a binary mixture undergoing the polymerization-induced phase separation. Using Flory–Huggins free energy density, the diagrams are calculated, where *ψ* = 1, *N*_1_ = 1, and from bottom to top, *N*_2_ = 1, 10, and 100.

Figure 1 presents the phase diagram of a monomer–solvent mixture, which has an upper critical solution temperature (UCST) and undergoes polymerization-induced phase separation (PIPS). A sample with the dimensionless initial average concentration *c*_0_* = 0.6 and the dimensionless temperature T* = 0.6 (black point) is initially located in the single-phase region, and the degrees of polymerization of the components are equal (*N*_1_ = *N*_2_ = 1); therefore, the phase diagram is symmetric. During polymerization, *N*_2_ increases, and the phase diagram shifts upward toward the higher temperature and solvent concentration and eventually passes the black point. Therefore, the sample is thrust into the unstable region of the phase diagram, and phase separation is induced. This approach is called polymerization-induced phase separation (PIPS).

**Figure 2:**
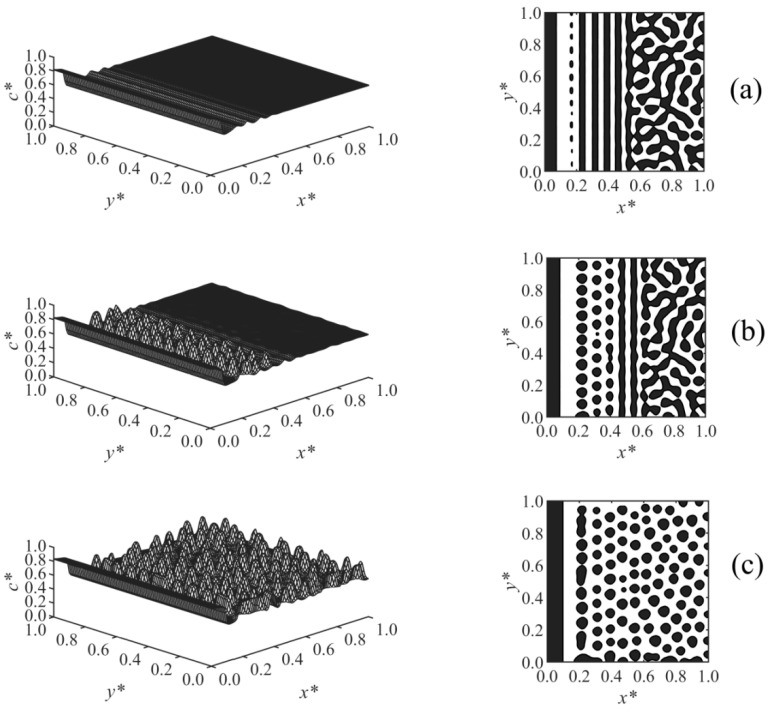
Spatial concentration distributions (first column) and phase-separated morphologies (second column) for Case 1 at the following times: (**a**) t* = 9.825 × 10^−5^, (**b**) t* = 9.835 × 10^−5^, and (**c**) t* = 9.85 × 10^−5^.

Figure 2 illustrates the time evolution of the surface-directed polymerization-induced phase separation morphology for Case 1 where the long-range surface potential was applied to the wall surface at x* = 0. The first column shows the spatial distributions of the dimensionless concentration of solvent c*, and the second column illustrates the phase-separated structures; the black areas are rich with respect to the solvent where c* > 0.6, and the white background is the polymer-rich region in which c* < 0.6; this has been followed for the next figures as well. Figure 2a show that at early times, the concentration waves appeared close to the interacting surface at x* = 0 where the surface potential dominates and attracts the solvent component. These waves were dampened by moving out into the bulk where phase separation has yet to begin. With increasing time, as shown in Figure 2b, the enrichment layer grew on the wall, while the adjacent stripes were broken down, and droplets appeared as a result of phase separation by spinodal decomposition in the region x* ≤ 0.42. However, phase separation has not yet begun farther in the bulk. Finally, the expected morphology by surface-directed spinodal decomposition (SDSD) was achieved at later times, as shown in Figure 2c, which consists of a wetting layer on the wall surface and a droplet-type morphology in the bulk. As Figure 2c illustrates, the second layer in the vicinity of the wall did not completely rupture, and the droplets formed in the bulk were aligned parallel to the wall up to x* ≈ 0.58, showing the dynamics of surface-directed spinodal decomposition (SDSD). However, farther into the bulk (x* > 0.58), the typical phase separation by spinodal decomposition morphology, i.e., the random droplet-type morphology, was observed. The enrichment layer is thicker as expected for a long-range surface potential compared to that obtained under the short-range surface potential in our previous work, where all the other conditions and parameters were the same [38]. 

**Figure 3:**
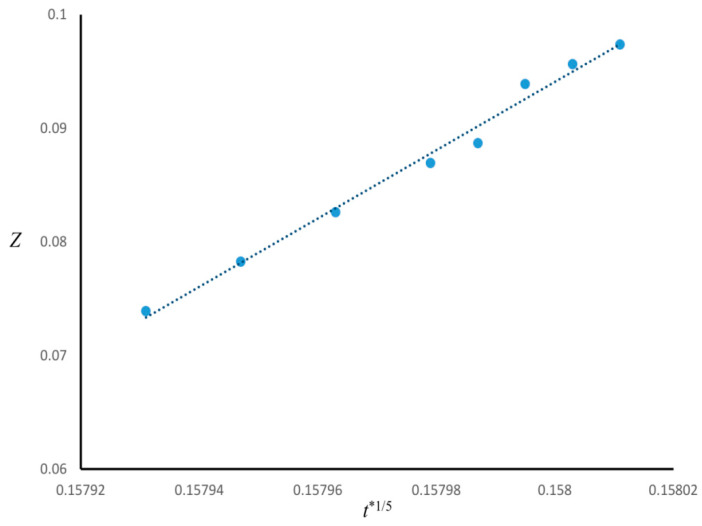
Growth of surface enrichment layer as a function of t*^1/5^ for Case 1.

The time evolution of the enrichment layer for Case 1 was investigated. Figure 3 is the plot of the thickness of the wetting layer *Z* versus t*^1/5^, indicating that *Z* ∝ t*^1/5^ (R^2^ ≈ 0.99). Since in Case 1 *n* = 3, the power-law function found for the growth of enrichment layer was consistent by the correlation (*Z* ∝ t* ^1/(*n*+2)^) found in the literature [19,20,21,22].

**Figure 4:**
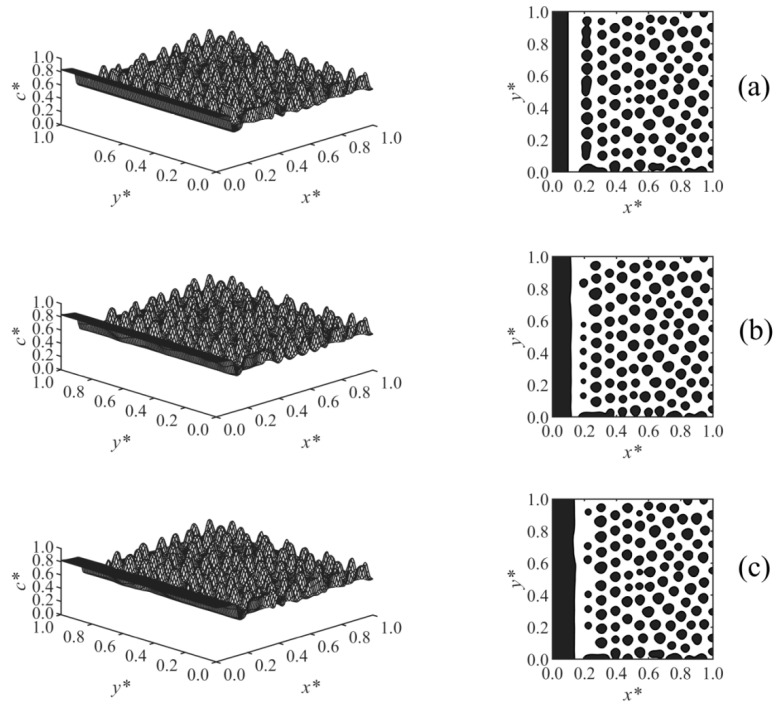
Spatial concentration distributions (first column) and phase-separated morphologies (second column) for Case 2 at t* = 9.85 × 10^−5^, at the following surface potential parameters: (**a**) *h*_1_ = 0.25, (**b**) *h*_1_ = 0.5, and (**c**) *h*_1_ = 2.

The effect of the surface potential parameter *h*_1_ on the phase-separated morphology was studied in Case 2, and the results are shown in Figure 4. A thicker enrichment layer is formed with increasing surface potential strength *h*_1_, as shown in Figure 4a–c. The results showed that at any time, Z ∝ *h*_1_^1/5^, which is consistent with the correlation Z ∝ *h*_1_^1/(*n*+2)^ reported in the literature for the early times before the crossover occurred [19,20,21,22]; *n* is the exponent of the surface potential, which *n* = 3 in Case 2. Although variations of *h*_1_ did not affect the morphology in the bulk, it does have an effect on the morphology in the second layer adjacent to the wall. This second layer was incompletely broken in Figure 4a with lower surface potential, but it was ruptured to droplets at the higher surface potential parameter in Figure 4b,c due to the stronger adsorption and formation of a thicker enriched layer on the wall.

**Figure 5:**
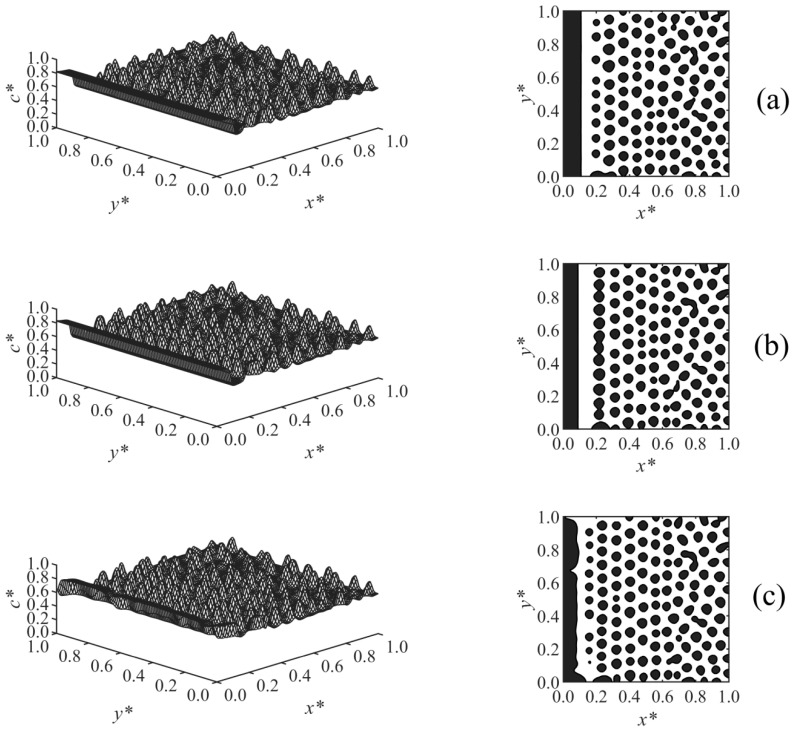
Spatial concentration distributions (first column) and phase-separated morphologies (second column) for Case 3 at t* = 9.8425 × 10^−5^, at the following exponents of surface potential: (**a**) *n* = 2, (**b**) *n* = 3, and (**c**) *n* = 4.

Figure 5 presents the results of Case 3 where the effect of the long-range surface potential exponent (*n*) in Equation (28) on the morphology development was investigated. The surface potential exponent determines the extent to which V* decays by moving away from the surface, where V* ∝ 1/*Z^n^*. By increasing the exponent from *n* = 2 (Figure 5a) to *n* = 3 (Figure 5b), the surface potential decayed at a smaller *Z*, which leads to the formation of a thinner enrichment layer. By increasing it further to *n* = 4 (Figure 5c), the surface potential V* becomes weaker, and a semi wetting layer was observed. This shows that the surface potential is weak compared to the strength of phase separation by spinodal decomposition, which causes the enrichment layer to break to a semi wetting substrate. This is due to the competing effects of the surface potential on the surface and phase separation by spinodal decomposition in the bulk.

**Figure 6:**
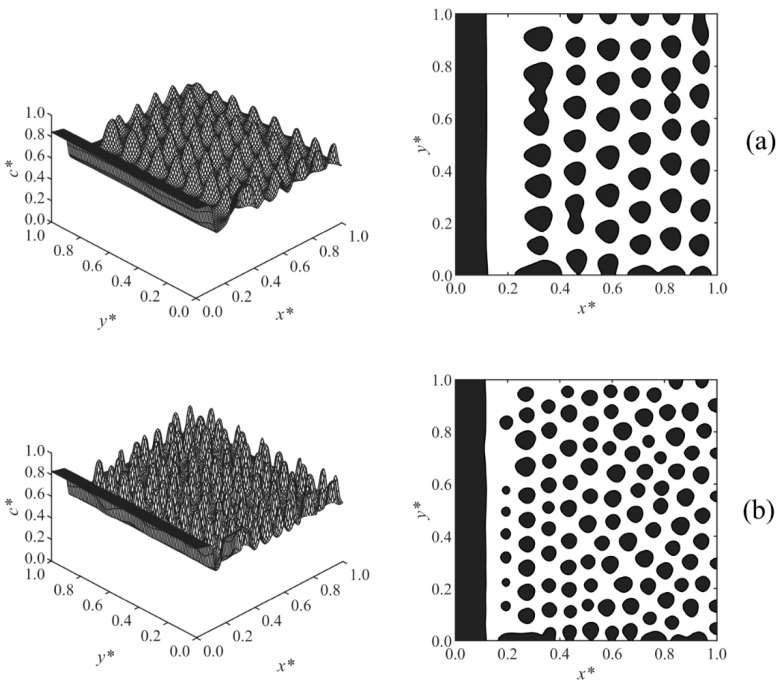
Spatial concentration distributions (first column) and phase-separated morphologies (second column) for Case 4 at the following dimensionless times and diffusion coefficients: (**a**) t* = 1.0025 × 10^−4^, *D* = 1 × 10^6^, (**b**) t* = 9.85 × 10^−5^, *D* = 3 × 10^6^.

Case 4, shown in Figure 6, investigated the effect of the diffusion coefficient on the long-range surface-directed polymerization-induced phase separation. A lower diffusion coefficient led to the formation of larger particles in the bulk as expected according to the diffusivity role in phase separation by spinodal decomposition [13,35,37,38,39,40]. Moreover, the droplets formed slowly, dominantly layered, and their circularity was far from unity in the low diffusivity condition (Figure 6a) compared to those in the high diffusivity condition (Figure 6b), which is consistent with the published study [13]. The reason is that at a low diffusion coefficient, phase separation occurs slowly, as the driving force is small. In addition, when the phase separation is induced by the polymerization, the quench depth is initially shallow, which slows down the phase separation by spinodal decomposition. Therefore, at a low diffusion coefficient, the surface potential effect was dominant and affected the phase-separated structure in the bulk by attracting the droplets. As a result, a pronounced layered structure (columns of droplets) forms, and the droplets resemble leftward pointers in Figure 6a instead of being circular, as shown in Figure 6b. The increase of diffusivity did not noticeably affect the thickness of the wetting layer, which indicates the significant strength of the long-range surface potential. The droplets could form and expand closer to the wall surface at a higher diffusivity (*D* = 3 × 10^6^), creating a depleted layer with respect to the solvent (shown in white) adjacent to the enriched layer (shown in black) that is thinner compared to that at a lower diffusion coefficient (*D* = 1 × 10^6^).

**Figure 7:**
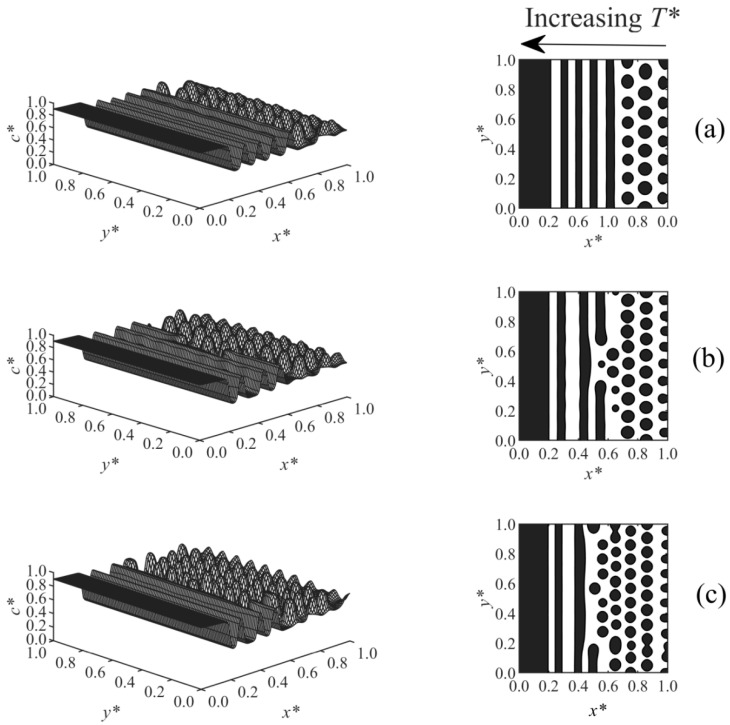
Spatial concentration distributions (first column) and phase-separated morphologies (second column) for Case 5 at the following dimensionless times and diffusion coefficients: (**a**) t* = 5.6 × 10^−5^, *D* = 6 × 10^5^, (**b**) t* = 5.55 × 10^−5^, *D* = 8 × 10^5^, and (**c**) t* = 5.525 × 10^−5^, *D* = 1 × 10^6^.

The effect of temperature gradient on the long-range surface-directed PIPS at different diffusivities was studied in Case 5, where a linear temperature gradient was applied to the domain in the same direction of the surface potential (i.e., T*_H_ and surface effect were applied at x* = 0). The morphologies obtained for Case 5 are illustrated in Figure 7a–c. The stripe morphology was observed in the high-temperature region, while the droplet-type morphology formed far from the interacting surface in the low temperature region, as expected [38]. The reason the stripes appeared and expanded into the bulk is that the high temperature side of the domain (at x* = 0) was thrust into the two-phase region of the phase diagram first, since the polymerization rate constant is larger at the high temperature (it is exponentially proportional to the temperature according to the Arrhenius’ equation) and leads to the fast upward movement of the phase diagram. Therefore, the surface-directed phase separation first occurred in the region close to the interacting surface, creating a wetting layer; in this region, there is a strong long-range surface effect with a weak spinodal decomposition effect due to the initial shallow quench. Therefore, a stripe morphology is initiated and expanded into the regions of lower temperatures. The stripe morphology is consistent with the multilayer structure formed experimentally by the SDSD of the binary alloys [5]. However, farther from the wall surface (i.e., in the bulk), the droplets gradually formed as the phase separation by spinodal decomposition could dominate over the surface effect. From Figure 7a–c, the diffusion coefficient increased, so the smaller droplets formed faster in Figure 7c (t* = 5.525×10^−5^), while larger droplets formed later under the lower diffusivities in Figure 7b (t* = 5.5×10^−5^) and Figure 7a (t* = 5.6×10^−5^). The droplets also expanded closer to the wall surface at the high diffusion coefficient (Figure 7c) compared to those at the lower diffusivities shown in Figure 7a,b, since the phase separation by spinodal decomposition was stronger at the high diffusivity and could prevent the stripes from forming. In the presence of a temperature gradient, the enrichment layer got slightly thinner with increasing diffusivity (Figure 7), while the increase of diffusivity did not change the thickness of the wetting layer when no temperature gradient was imposed (Figure 6); it shows that the temperature gradient contributed to the effect of diffusivity on the morphology development.

**Figure 8:**
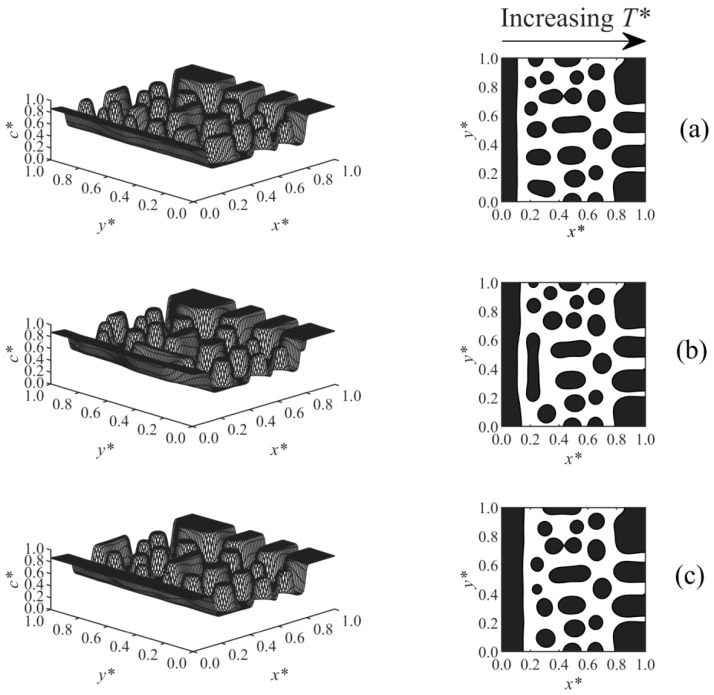
Spatial concentration distributions (first column) and phase-separated morphologies (second column) for Case 6 at t* = 5.75 × 10^−5^ and the following surface potential parameters: (**a**) *h*_1_ = 0.5, (**b**) *h*_1_ = 1, and (**c**) *h*_1_ = 2.

Figure 8 shows Case 6, which considers the effect of a linear temperature gradient on the surface-directed spinodal decomposition morphology under various surface potential parameters, where T*_L_ and the surface effect wereapplied at x* = 0, and T*_H_ was imposed at x* = 1. As the results illustrate, large droplets formed in the high-temperature region (right-hand side of the domain), since this region was thrust into the unstable region of the phase diagram and underwent the early, intermediate, and even late stages of phase separation earlier than the low-temperature region, which is in good agreement with the previous studies [36,37,38,54]. Therefore, larger and more concentrated droplets appeared in the high-temperature region, while smaller and less concentrated particles formed at the lower-temperature sections. The anisotropic morphology in the bulk is consistent with the anisotropic structure of the polymer membranes fabricated by phase separation via spinodal decomposition under the temperature/concentration gradients [51,55,56]. The wetting layer formed close to the wall at the T*_L_ side. Imposing a stronger surface potential (from Figure 8a–c) created a thicker enriched layer.

## 4. Conclusions 

The long-range surface directed polymerization-induced phase separation of a monomer–solvent sample undergoing self-condensation polymerization was investigated. The Cahn–Hilliard and Flory–Huggins free energy theories were used to model the phase separation by spinodal decomposition. The results showed that the wetting layer formed close to the interacting surface, while droplets appeared in the bulk. The thickness of the wetting layer versus the surface potential parameter and time obeyed the power-law function, where the exponent was equal to 1/5 for both of them. This is consistent with the literature. The increase of diffusivity led to faster and smaller droplets formed in addition to the thinner depletion layer. A temperature gradient led to the formation of an anisotropic droplet-type morphology in the bulk if it was imposed in the opposite direction of the surface potential, while a stripe morphology was observed under the temperature gradient imposed in the same direction of surface potential. 

## Figures and Tables

**Table 1:** The parameters and conditions considered for each case. For all cases, *c*_0_* = 0.6, *σ* = 99, *γ* = 1, *g* = −0.5, E_a_* = 10, and *ψ* = 1.

Case	Conditions	T*	*D*	A*	*h*_1_	*n*
1	Long-range surface potential at x* = 0	0.6	3 × 10^6^	10^11^	0.25	3
2	Long-range surface potential at x* = 0	0.6	3 × 10^6^	10^11^	(a) 0.25 (b) 0.5 (c) 2	3
3	Long-range surface potential at x* = 0	0.6	3 × 10^6^	10^11^	0.25	(a) 2 (b) 3 (c) 4
4	Long-range surface potential at x* = 0	0.6	(a) 1 × 10^6^ (b) 3 × 10^6^	10^11^	0.5	3
5	Long-range surface potential at x* = 0 ΔT* T*_H_ at x* = 0 T*_L_ at x* = 1	0.595–0.6	(a) 6 × 10^5^ (b) 8 × 10^5^ (c) 1 × 10^6^	2 × 10^11^	0.5	3
6	Long-range surface potential at x* = 0 ΔT* T*_L_ at x* = 0 T*_H_ at x* = 1	0.595–0.6	6 × 10^5^	2 × 10^11^	(a) 0.5 (b) 1 (c) 2	3

## Data Availability

All data in this study were generated by our research group, and they are included in this article.
